# Complete mitochondrial genome of the house bat *Pipistrellus abramus* (Mammalia: Chiroptera) from Korea

**DOI:** 10.1080/23802359.2017.1365644

**Published:** 2017-08-17

**Authors:** Ji Young Kim, Hye Ri Kim, Sang Jin Lim, Hyun Ju Kim, Jae Youl Cho, Yung Chul Park

**Affiliations:** a Division of Forest Science, College of Forest and Environmental Sciences, Kangwon National University, Republic of Korea;; b Ecosystem Research Division, National Park Research Institute, Korea National Park Service, Wonju, Republic of Korea;; c Microbial Safety Team, National Institute of Agricultural Sciences, Rural Development Administration, Wanju, Republic of Korea;; d Department of Genetic Engineering, Sungkyunkwan University, Suwon, Republic of Korea

**Keywords:** Complete mitogenome, Korean house bat, *Pipistrellus abramus*

## Abstract

We determined and annotated the complete mitogenome of the house bat *Pipistrellus abramus* (Chiroptera: Vespertilionidae) from Korea. The complete mitogenome is a circular molecule of 17,236 bp in length, including 13 protein-coding genes, 2 ribosomal RNA genes, 22 transfer RNA genes, and 2 non-coding regions (L-strand replication origin and control region). The mitogenome is AT-biased, with a nucleotide composition of 33.7% A, 29.9% T, 23.2% C, and 13.2% G. The phylogenetic analysis revealed that the house bat *P. abramus* from Korea is well grouped with that from Japan and placed within the genus *Pipistrellus* clade, which has the noctule bat *Nyctalus* as sister clade.

The house bats of *Pipistrellus abramus*, known as a species of vesper bat, are wildly distributed across East Asia, from China and Taiwan into the Ussuri region (Russia and China), the Korean Peninsula, and Japan (Won 2004; Bates and Tsytsulina 2008).

We sequenced and annotated a mitogenome of *P. abramus* from Korea. A wing membrane tissue sample for genomic DNA extraction was collected from a bat individual caught around an agricultural region in Odaesan National Park (N37 42 42.8, E128 35 57.6), South Korea. The voucher specimen (VEPIAB-1) was deposited in the Wildlife and Fish Conservation Center of the Institute of Forest Science, Kangwon National University. Genomic DNA extraction, PCR, and gene annotation were conducted according to the previous studies (Yoon et al. 2013; Jeon and Park 2015; Rahman et al. 2016). Previously published mitogenomes of Indian *P. coromandra* (KP688404) and Japanese *P. abramus* (NC_005436) were used as references for gene annotation and primer design for PCR amplification of the Korean *P. abramus* mitogenome. Phylogenetic tree was constructed using maximum-likelihood (ML) procedures implemented in MEGA6 (Tamura et al. 2013).

The complete mitogenome (KX355640) of the Korean house bat *P. abramus* contains total 17,236 bp in length, which consists of a control region (one D-loop region) and a conserved set of 37 genes including 13 protein-coding genes (PCGs), 22 tRNA genes, and 2 ribosomal RNA genes (*12S rRNA* and *16S rRNA*). The mitogenome is AT-biased, with a nucleotide composition of 33.7% A, 29.9% T, 23.2% C, and 13.2% G.

Total length of the 22 tRNA genes is 1517 bp and their average length is 69 ± 3.3 bp, ranging from 59 bp (*tRNA^Ser(AGY)^*) to 74 bp (*tRNA^Phe^*, *tRNA^Gln^*, and *tRNA^Leu(UUR)^*). Lengths of the two rRNA genes and control region are 955 bp (*12S rRNA*), 1567 bp (*16S rRNA*), and 928 bp (control regions), respectively. Total length of 13 PCGs is 11,379 bp, with the exclusion of stop codons (30 bp), which encode 3793 amino acids. Mitochondrial PCGs of *P. abramus* use the three kinds of start codon. ATG is the most common start codon, which is used in 11 PCGs, but the start codons ATT and ATA are used only once in *Nd3* and *Nd5*, respectively. The incomplete stop codons are used for termination of five PCGs (TA − for *Nd1* and T − for *Nd2, Nd3, Nd4,* and *Cox3*). AGA is used only once as a stop codon for *Cytb*. TAA is most common stop codon, which is used for termination of all the other seven PCGs. The replication origin *O_L_*is 35 bp in size and located between *tRNA^Asn^* and *tRNA^Cys^* within the WANCY tRNA cluster as seen in most vertebrates (Kim and Park 2012; Yoon et al. 2013; Nam et al. 2015; Rahman et al. 2016).

The phylogenetic analysis revealed that the house bat *P. abramus* from Korea is well grouped with that from Japan and placed within the genus *Pipistrellus* clade, which has the noctule bat *Nyctalus* as sister clade (Figure 1).

**Figure 1.**
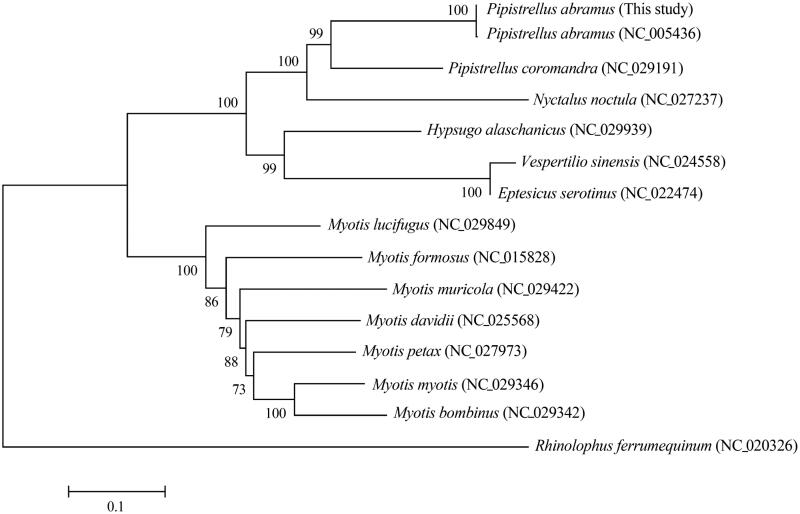
The phylogenetic relationship of *P. abramus* and its allied species inferred from maximum-likelihood analysis based on mitogenome sequences. The ML tree was generated using the GTR + G + I model, and the robustness of the tree was tested with 1000 bootstrap. The numbers on the branches indicate bootstrap values.
